# Preoperative, intraoperative, and postoperative complications in orthognathic surgery: a systematic review

**DOI:** 10.1007/s00784-015-1452-1

**Published:** 2015-03-26

**Authors:** M. Jędrzejewski, T. Smektała, K. Sporniak-Tutak, R. Olszewski

**Affiliations:** 1 Department of Dental Surgery, Pomeranian Medical Uniwersity, ul. Powstańców Wielkopolskich 72, 70-111 Szczecin, Poland; 2 Department of Maxillofacial Surgery, Pomeranian Medical University, ul. Powstańców Wielkopolskich 72, 70-111 Szczecin, Poland; 3 Department of Oral and Maxillofacial Surgery, Université catholique de Louvain, 10 Av. Hippocrate, 1200 Brussels, Belgium

**Keywords:** Orthognathic surgery, Complications, Systematic review, Le Fort I, BSSO

## Abstract

**Objectives:**

The aim of this study was to determine whether orthognathic surgery is associated with any complications, and what type of complications may occur.

**Materials and methods:**

Data were obtained using PubMed (MEDLINE), ISI Web of Knowledge, Ovid, Cochrane Library, Embase Library, and an additional manual search. The titles and abstracts of the electronic search results were screened and evaluated by two observers for eligibility according to the inclusion and exclusion criteria.

**Results:**

A total of 1924 articles were identified, and we retained 44 articles for the final analysis. The Prisma diagram flowchart demonstrates our selection scheme. For the purpose of this study, the Cochrane data extraction form was modified. One review author extracted data from the included studies, and the second author checked all of the forms. The hierarchy of evidence classification from the UK NHS Centre for Reviews and Dissemination was used to assess the level of evidence for the retrieved studies.

**Conclusions:**

An evaluation of the obtained studies revealed the existence of a large number of varied complications associated with orthognathic surgery procedures.

**Clinical relevance:**

Oral and maxillofacial surgeons, orthodontists, and the surgical team need to prevent such complications during preoperative, intraoperative, and postoperative periods to increase the safety of orthognathic surgery procedures. This review was registered on http://www.crd.york.ac.uk/PROSPERO as CRD42013004711.

**Electronic supplementary material:**

The online version of this article (doi:10.1007/s00784-015-1452-1) contains supplementary material, which is available to authorized users.

## Introduction

Orthognathic surgery procedures are frequently used to correct skeletal angle class II and III deformities, dentomaxillofacial deformities, mandibular laterognathia, and maxillofacial asymmetries [1–4]. As with any surgical procedure, various preoperative, intraoperative, and postoperative complications may occur. Two systematic reviews of orthognathic surgery complications have been previously published on the following topics [1, 5]: (1) blood loss following orthognathic surgery [5] and (2) complications during bilateral sagittal split osteotomy (BSSO) [1]. The systematic review on blood loss presents some methodological pitfalls. The research equation used in this study to identify relevant articles resulted in the identification of only seven articles. In addition, the data investigation was performed by only one reviewer, and the assessment of the potential risk of bias was unclear. The article investigated a narrow group of possible complications. The methodology of the second systematic review [1] was well designed and provided reliable conclusions. However, it was limited to the description of complications associated with only one type of orthognathic surgery procedure (BSSO). Therefore, we wanted to provide an extensive systematic review of complications in orthognathic surgery according to strict requirements of evidence-based medicine. The null hypothesis was that complications are inherent to orthognathic surgery procedures. The aim of our study was to answer to the clinical question asking what are the complications associated with orthognathic surgery.

## Materials and methods

Protocol and registration: This review was registered on http://www.crd.york.ac.uk/PROSPERO as CRD42013004711.

Eligibility criteria: No publication date restrictions were imposed. All systematic reviews, randomized controlled trials, clinical trials were considered. English, German, French, or Polish language articles were included in the search. Patients of any age who had any orthognathic surgery procedure were evaluated in this review.

Information sources: The first literature search for studies of complications in orthognathic surgery was executed using PubMed (MEDLINE), ISI Web of Knowledge, Ovid, and the Cochrane Library on 18.01.2013. The second search using Embase Library was performed and updated on 28.02.2013 with the assistance of a Senior Librarian. The additional search with the same search equation was performed in Google Scholar and by browsing references of acquired studies on 24.03.2013. The last update of our search using PubMed (MEDLINE), ISI Web of Knowledge, and Ovid was performed on 17.02.2015.

Search: The search equation consisted of 4 primary keywords in combination with 59 secondary keywords (Table 1). The search results obtained from PubMed (MEDLINE) and Embase Library databases are depicted in Tables 2 and 3.

**Table 1:** Primary and secondary keywords used for the systematic review

Primary keywords (*n* = 4)	Secondary keywords (*n* = 59)
(complication* OR failur* OR harms) AND orthognathic	hemorrhage [MeSH Terms] OR hemorrhage [TW] OR bleeding [TW] OR orthognathic surgery [MeSH Terms] [TW] OR excessive blood loss [TW] OR neurosensory disturbance [TW] OR facial nerve [TW] OR facial nerve paralysis [TW] OR cranial nerve injuries [MeSH Terms] OR bad split* [TW] OR malocclusion* [TW] OR depression* [TW] OR depression [MeSH Terms] OR prognathism [TW] OR prognathism [MeSH Terms] OR retrognathism [MeSH Terms] OR micrognathism [TW] OR micrognathism [MeSH Terms] OR orthognathic surgical procedures [MeSH Terms] OR orthognathic surgical procedures [TW] OR osteotomy, Le Fort [MeSH Terms] OR Le Fort [TW] OR osteotomy, sagittal split ramus [MeSH Terms] OR osteotomy, sagittal split ramus [TW] OR soft tissue injuries [MeSH Terms] OR soft tissue injuries [TW] OR soft tissue infection [MeSH Terms] OR hard tissue [TW] OR inflammation [MeSH Terms] OR infection [TW] OR bone resorption [MeSH Terms] OR resorption [TW] OR diagnosis [MeSH Terms] OR mortality [MeSH Terms] OR condylar resorption [TW] OR condylar reposition [TW] OR condylar resection [TW] OR TMJ ankylosis [TW] OR joint diseases [MeSH Terms] OR joint diseases [MeSH Terms] OR TMJ degeneration [TW] OR preoperative [TW] OR postoperative [TW] OR screw [TW] OR plates [TW] OR lingual nerve [TW] OR teeth [TW] OR teeth [TW] OR roots [TW] OR osteosynthesis [TW] OR morbidity [TW] OR inferior alveolar nerve [TW] OR mental disorder [TW] OR anatomy [TW] OR CT, CBCT [MeSH Terms] OR airways [MeSH Terms] OR face bow [TW] OR plaster cast [TW]

**Table 2:** Search strategy equation for PubMed (Medline) database

Set	Search terms	Results
#1	(complication* OR failur* OR harms) AND orthognathic	430
#2	hemorrhage OR hemorrhage OR bleeding OR orthognathic surgery OR excessive blood loss OR neurosensory disturbance OR facial nerve OR facial nerve paralisis OR cranial nerve injuries OR bad split* OR malocclusion OR depression* OR depression OR prognathism OR prognathism OR retrognathism OR micrognathism OR micrognathism OR orthognathic surgical procedures OR orthognathic surgical procedures OR osteotomy, Le Fort OR Le Fort OR osteotomy, sagittal split ramus OR osteotomy, sagittal split ramus OR soft tissue injuries OR soft tissue injuries OR soft tissue infection OR hard tissue OR inflammation OR infection OR bone resorption OR resorption OR diagnosis OR mortality OR condylar resorption OR condylar reposition OR condylar resection OR TMJ ankylosis OR joint diseases OR joint diseases OR TMJ degeneration OR preoperative OR postoperative OR screw OR plates OR lingual nerve OR teeth OR teeth OR roots OR osteosynthesis OR morbidity OR inferior alveolar nerve OR mental disorder OR anatomy OR CT, CBCT OR airways OR face bow OR plaster cast	3161370
#3	#1 AND #2	428

**Table 3:** Search strategy equation for Embase database

Set	Search terms	Results
#1	orthognathic AND ([article]/lim OR [article in press]/lim) AND ([english]/lim OR [french]/lim OR [german]/lim OR [polish]/lim) AND [humans]/lim AND [abstracts]/lim AND [embase]/lim AND (hemorrhage AND ([article]/lim OR [article in press]/lim) AND ([english]/lim OR [french]/lim OR [german]/lim OR [polish]/lim) AND [humans]/lim AND [abstracts]/lim AND [embase]/lim OR (bleeding AND ([article]/lim OR [article in press]/lim) AND ([english]/lim OR [french]/lim OR [german]/lim OR [polish]/lim) AND [humans]/lim AND [abstracts]/lim AND [embase]/lim) OR (orthognathic AND surgery AND ([article]/lim OR [article in press]/lim) AND ([english]/lim OR [french]/lim OR [german]/lim OR [polish]/lim) AND [humans]/lim AND [abstracts]/lim AND [embase]/lim) OR (excessive AND blood AND loss AND ([article]/lim OR [article in press]/lim) AND ([english]/lim OR [french]/lim OR [german]/lim OR [polish]/lim) AND [humans]/lim AND [abstracts]/lim AND [embase]/lim) OR (neurosensory AND disturbance AND ([article]/lim OR [article in press]/lim) AND ([english]/lim OR [french]/lim OR [german]/lim OR [polish]/lim) AND [humans]/lim AND [abstracts]/lim AND [embase]/lim) OR (facial AND nerve AND ([article]/lim OR [article in press]/lim) AND ([english]/lim OR [french]/lim OR [german]/lim OR [polish]/lim) AND [humans]/lim AND [abstracts]/lim AND [embase]/lim) OR (facial AND nerve AND paralisis AND ([article]/lim OR [article in press]/lim) AND ([english]/lim OR [french]/lim OR [german]/lim OR [polish]/lim) AND [humans]/lim AND [abstracts]/lim) OR (bad AND split AND ([article]/lim OR [article in press]/lim) AND ([english]/lim OR [french]/lim OR [german]/lim OR [polish]/lim) AND [humans]/lim AND [abstracts]/lim AND [embase]/lim) OR (bad AND splits AND ([article]/lim OR [article in press]/lim) AND ([english]/lim OR [french]/lim OR [german]/lim OR [polish]/lim) AND [humans]/lim AND [abstracts]/lim AND [embase]/lim) OR (malocclusion AND ([article]/lim OR [article in press]/lim) AND ([english]/lim OR [french]/lim OR [german]/lim OR [polish]/lim) AND [humans]/lim AND [abstracts]/lim AND [embase]/lim) OR (depression AND ([article]/lim OR [article in press]/lim) AND ([english]/lim OR [french]/lim OR [german]/lim OR [polish]/lim) AND [humans]/lim AND [abstracts]/lim AND [embase]/lim) OR (prognathism AND [article in press]/lim AND ([english]/lim OR [french]/lim OR [german]/lim OR [polish]/lim) AND [humans]/lim AND [abstracts]/lim AND [embase]/lim) OR (micrognathism AND ([article]/lim OR [article in press]/lim) AND ([english]/lim OR [french]/lim OR [german]/lim OR [polish]/lim) AND [humans]/lim AND [abstracts]/lim AND [embase]/lim) OR (orthognathic AND surgical AND procedures AND ([article]/lim OR [article in press]/lim) AND ([english]/lim OR [french]/lim OR [german]/lim OR [polish]/lim) AND [humans]/lim AND [abstracts]/lim AND [embase]/lim) OR (le AND fort AND ([article]/lim OR [article in press]/lim) AND ([english]/lim OR [french]/lim OR [german]/lim OR [polish]/lim) AND [humans]/lim AND [abstracts]/lim AND [embase]/lim) OR (osteotomy, AND sagittal AND split AND ramus AND ([article]/lim OR [article in press]/lim) AND ([english]/lim OR [french]/lim OR [german]/lim OR [polish]/lim) AND [humans]/lim AND [abstracts]/lim AND [embase]/lim) OR (soft AND tissue AND injuries AND ([article]/lim OR [article in press]/lim) AND ([english]/lim OR [french]/lim OR [german]/lim OR [polish]/lim) AND [humans]/lim AND [abstracts]/lim AND [embase]/lim) OR (hard AND tissue AND ([article]/lim OR [article in press]/lim) AND ([english]/lim OR [french]/lim OR [german]/lim OR [polish]/lim) AND [humans]/lim AND [abstracts]/lim AND [embase]/lim) OR (infection AND ([article]/lim OR [article in press]/lim) AND ([english]/lim OR [french]/lim OR [german]/lim OR [polish]/lim) AND [humans]/lim AND [abstracts]/lim AND [embase]/lim) OR (resorption AND ([article]/lim OR [article in press]/lim) AND ([english]/lim OR [french]/lim OR [german]/lim OR [polish]/lim) AND [humans]/lim AND [abstracts]/lim AND [embase]/lim) OR (condylar AND resorption AND ([article]/lim OR [article in press]/lim) AND ([english]/lim OR [french]/lim OR [german]/lim OR [polish]/lim) AND [humans]/lim AND [abstracts]/lim) OR (condylar AND reposition AND ([article]/lim OR [article in press]/lim) AND ([english]/lim OR [french]/lim OR [german]/lim OR [polish]/lim) AND [humans]/lim AND [abstracts]/lim AND [embase]/lim) OR (condylar AND resection AND ([article]/lim OR [article in press]/lim) AND ([english]/lim OR [french]/lim OR [german]/lim OR [polish]/lim) AND [humans]/lim AND [abstracts]/lim AND [embase]/lim) OR (tmj AND ankylosis AND ([article]/lim OR [article in press]/lim) AND ([english]/lim OR [french]/lim OR [german]/lim OR [polish]/lim) AND [humans]/lim AND [abstracts]/lim AND [embase]/lim) OR (tmj AND degeneration AND ([article]/lim OR [article in press]/lim) AND ([english]/lim OR [french]/lim OR [german]/lim OR [polish]/lim) AND [humans]/lim AND [abstracts]/lim AND [embase]/lim) OR (preoperative AND ([article]/lim OR [article in press]/lim) AND ([english]/lim OR [french]/lim OR [german]/lim OR [polish]/lim) AND [humans]/lim AND [abstracts]/lim AND [embase]/lim) OR (postoperative AND ([article]/lim OR [article in press]/lim) AND ([english]/lim OR [french]/lim OR [german]/lim OR [polish]/lim) AND [humans]/lim AND [abstracts]/lim AND [embase]/lim) OR (screw AND ([article]/lim OR [article in press]/lim) AND ([english]/lim OR [french]/lim OR [german]/lim OR [polish]/lim) AND [humans]/lim AND [abstracts]/lim AND [embase]/lim) OR (plates AND ([article]/lim OR [article in press]/lim) AND ([english]/lim OR [french]/lim OR [german]/lim OR [polish]/lim) AND [humans]/lim AND [abstracts]/lim AND [embase]/lim) OR (lingual AND nerve AND ([article]/lim OR [article in press]/lim) AND ([english]/lim OR [french]/lim OR [german]/lim OR [polish]/lim) AND [humans]/lim AND [abstracts]/lim AND [embase]/lim) OR (teeth AND ([article]/lim OR [article in press]/lim) AND ([english]/lim OR [french]/lim OR [german]/lim OR [polish]/lim) AND [humans]/lim AND [abstracts]/lim AND [embase]/lim) OR (roots AND ([article]/lim OR [article in press]/lim) AND ([english]/lim OR [french]/lim OR [german]/lim OR [polish]/lim) AND [humans]/lim AND [abstracts]/lim AND [embase]/lim) OR (osteosynthesis AND ([article]/lim OR [article in press]/lim) AND ([english]/lim OR [french]/lim OR [german]/lim OR [polish]/lim) AND [humans]/lim AND [abstracts]/lim AND [embase]/lim) OR (morbidity AND ([article]/lim OR [article in press]/lim) AND ([english]/lim OR [french]/lim OR [german]/lim OR [polish]/lim) AND [humans]/lim AND [abstracts]/lim AND [embase]/lim) OR (inferior AND alveolar AND nerve AND ([article]/lim OR [article in press]/lim) AND ([english]/lim OR [french]/lim OR [german]/lim OR [polish]/lim) AND [humans]/lim AND [abstracts]/lim AND [embase]/lim) OR (mental AND disorder AND ([article]/lim OR [article in press]/lim) AND ([english]/lim OR [french]/lim OR [german]/lim OR [polish]/lim) AND [humans]/lim AND [abstracts]/lim AND [embase]/lim) OR (anatomyAND ([article]/lim OR [article in press]/lim) AND ([english]/lim OR [french]/lim OR [german]/lim OR [polish]/lim) AND [humans]/lim AND [abstracts]/lim AND [embase]/lim) OR (face AND bow AND ([article]/lim OR [article in press]/lim) AND ([english]/lim OR [french]/lim OR [german]/lim OR [polish]/lim) AND [humans]/lim AND [abstracts]/lim AND [embase]/lim) OR (plaster AND cast AND ([article]/lim OR [article in press]/lim) AND ([english]/lim OR [french]/lim OR [german]/lim OR [polish]/lim) AND [humans]/lim AND [abstracts]/lim AND [embase]/lim) OR 'bleeding'/exp OR 'orthognathic surgery'/exp OR 'cranial nerve injury'/exp OR 'depression'/exp OR 'prognathia'/exp OR 'retrognathia'/exp OR 'micrognathia'/exp OR 'maxilla osteotomy'/exp OR 'sagittal split ramal osteotomy'/exp OR 'soft tissue injury'/exp OR 'soft tissue infection'/exp OR 'inflammation'/exp OR 'osteolysis'/exp OR 'diagnosis'/exp OR 'mortality'/exp OR 'temporomandibular joint disorder'/exp OR 'cone beam computed tomography'/exp) AND (harms AND ([article]/lim OR [article in press]/lim) AND ([english]/lim OR [french]/lim OR [german]/lim OR [polish]/lim) AND [humans]/lim AND [abstracts]/lim AND [embase]/lim OR (failur AND ([article]/lim OR [article in press]/lim) AND ([english]/lim OR [french]/lim OR [german]/lim OR [polish]/lim) AND [humans]/lim AND [abstracts]/lim AND [embase]/lim) OR (complication AND ([article]/lim OR [article in press]/lim) AND ([english]/lim OR [french]/lim OR [german]/lim OR [polish]/lim) AND [humans]/lim AND [abstracts]/lim AND [embase]/lim))	246

**Fig. 1:**
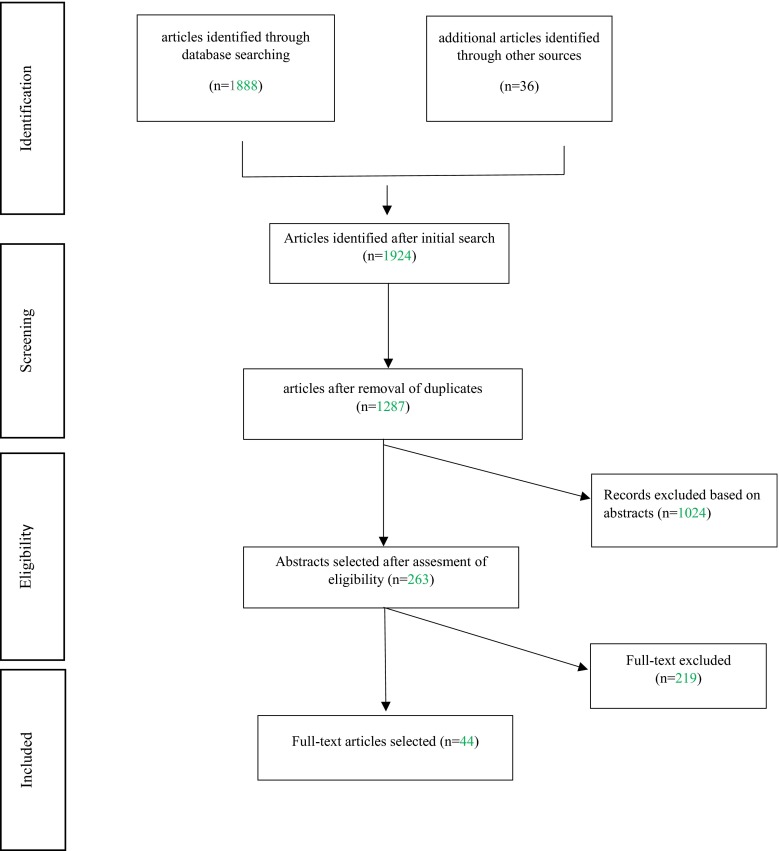
PRISMA diagram flowchart

Study selection: The titles and abstracts obtained from the electronic search were screened and evaluated in an unblinded standardized manner by two observers for eligibility according to the inclusion and exclusion criteria (Table 4). Studies not meeting the inclusion criteria were excluded from further evaluation. Any discrepancies in the selection were settled through discussion. A total of 1888 references from the automatic database searches and 36 supplementary references after a manual search were included for evaluation. Of the 1924 articles initially identified, 1287 articles (100 % of those evaluated) remained after the automatic rejection of duplicates in the EndNote X5 reference manager. After verification with respect to inclusion/exclusion criteria, 1024 articles (79.56 %) were excluded. A total of 263 full-text articles (25.68 %) were read in their entirety. A total of 219 full-text articles (83.27 %) met the criteria of our review, but the final number of included articles was 44. The included articles contained five randomized controlled trials (11.36 %) and 39 clinical trials (88.64 %). A Prisma diagram flowchart presents the selection scheme (Fig. 1).

**Table 4:** Inclusion and exclusion criteria used for data search

Inclusion criteria	Exclusion criteria
-methodogical design: systematic reviews, randomized controlled trials, clinical trials, -Languages: English, German or French or Polish -Human studies only -In vivo studies -No limit time	-Studies other than: systematic reviews, randomized clinical trials, clinical trials -No authors -Languages other than: English, German or French or Polish -Animal studies -In vitro studies -Irrelevant to orthognathic surgery

Data collection process: For the purpose of this study, the Cochrane data extraction form was modified (based on the Cochrane Consumers and Communication Review Group’s data extraction template). One review author extracted data from the included studies, and the second author checked all of the forms. Information obtained from the data extraction forms were as follows: the characteristics of the participants (age, principal health problem, and severity of disease), the type of intervention, the outcomes of the undertaken treatment, and the design of the reported study form.

Risk of bias in individual studies: To provide the most reliable evidence, a critical appraisal of all included randomized controlled trials (RCTs) and clinical trials (CTs) was performed (Online Resources 1–2). The Cochrane Collaboration Tool for the assessment of risk of bias was used to conduct this assessment. The overall judgment was assessed as high or unclear risk when one or more key domains were assessed as high or unclear. The low-risk judgment was assigned when all key domains were assessed as low risk. The PRISMA checklist is included in Online Resource 3.

## Results

Most of the references searched in the databases constituted case reports, case series, reviews, or comparative studies (79.91 %). Clinical trials (CT) represented a smaller group of studies (17.81 %); additionally, only a few of the clinical studies were randomized (RCT) (2.28 %). With respect to the time of exposure, complications occurred preoperatively, intraoperatively, and postoperatively. RCTs and CTs are crucial to evidence-based medicine as the most reliable source of information; therefore, only these types of studies were included in our evaluation. RCTs and CTs searched during our review presented the following complications: nerve injury/sensitivity alteration (50.00 %), temporomandibular joint (TMJ) disorders or impairment (13.64 %), hemorrhage (9.09 %), auditory tube function and hearing problems (6.82 %), infection (6.82 %), bad split (4.55 %), nonunion of osteotomy gap (4.55 %), skeletal relapse (4.55 %), septum deviation (2.28 %), bone necrosis (2.28 %), soft tissue injuries (2.28 %), positional vertigo (2.28 %), dental complications (2.28 %), postoperative swelling (2.28 %), and psychological depression (2.28 %).

A critical appraisal of all included randomized controlled trials [6–10] and clinical trials [11–49] was performed to provide the most reliable evidence. All of the RCTs were designated as having unclear risk assessment (Online Resource 1). Of all of the clinical trials, only three studies [27, 30, 41] were assessed as low risk, one third was classified as high risk [11–14, 16, 21–23, 34, 42, 43], and the remaining 20 articles exhibited unclear risk [15, 17–20, 24–26, 28, 29, 31–33, 35–40, 44–49] (Online Resource 2). These results demonstrated that only 3 out of 44 assessed studies [27, 30, 41] met all of the requirements of our critical appraisal. The most common reason for an unclear or high-risk designation was the unblinded evaluation of clinical outcomes.

## Discussion

The first records of the use of Le Fort I osteotomy and bilateral sagittal split mandibular osteotomy (BSSO) procedures for the correction of midfacial deformities were described in the 1920s [50] and in 1953 [51], respectively. The earliest article describing complications associated with such a procedure dates back to 1979 [52]. The rate of reported complications has gradually increased with time, from only one study in 1979 to 14 studies in 2012, as orthognathic surgery has become more widely accepted, and is now a frequently performed surgical method for correcting maxillomandibular dysmorphoses. However, the total number of complications might be underestimated because surgeons may be unable to easily report the complications due to their own professional obligations and involvement.

According to the articles obtained in our search, the most commonly reported complication was cranial nerve injury/sensitivity alteration (50.00 %). Following orthognathic surgery, patients may encounter laceration or disruption as also stretching of the cranial nerves, especially the inferior alveolar nerve (IAN) during BSSO. Neurophysiologic examination with electroneuromyography enables the exact classification of nerve injury into either the axonal or demyelinating type, which allows the accurate prediction of recovery and the risk of neuropathic pain [29]. Demyelinating nerve injury recovers completely within 2 to 4 months along with remyelination, and it very seldom induces neuropathic pain. On the other hand, axonal injury often recovers incompletely, and slowly, over months or even years, and entails a higher risk of pain developement [29]. The subjective symptoms of altered sensation were classified according to the general sensory system dysfunction classification [29] into three categories: normal symptoms (nerves with no subjective alteration), negative sensory symptoms (hypoesthesia), and positive sensory symptoms (parasthesia, dysasthesia, and/or pain). Subjective symptoms of sensory alteration are more important after axonal rather than with demyelinating injuries [29]. Methods for testing sensory nerve function can be divided as follows: qualitative (touch sensation, sharp/blunt test, cold sensation, and hot sensation) and quantitative methods (localization test, two point static, and dynamic test) [31]. Researchers usually measured sensory impairment immediately after surgery, after 3, 6 months, and after 1 year. Philips et al. reported that immediately after surgery almost all patients reported altered sensation [9]. Most cases of paresthesia resolved within 1 year, but not all [37]. Henzelka et al. found that approximately 3 % of patients may suffer from paresthesia even 1 year after surgery. The same authors found a significantly higher prevalence of paresthesia on the left side [37]. In their opinion, a higher prevalence of IAN disturbance on the left side suggests the importance of asymmetry in the relationship between the surgeon and the operative field or asymmetry of the surgical procedure. Further risk factors for IAN injury and impairment are the following: (1) patient’s age; (2) length of procedure; (3) experience of the surgeon; (4) the type of procedure (ILRO-inverted L ramus osteotomy seems to be a better choice than the BSSO method); (5) mandibular advancement >10 mm; (6) type of fixation (bicortical fixation seems to be a risk factor for nerve injury or compression); (7) the surgical space on the medial side of the mandibular ramus and the subsequent manipulation of the IAN in that region; and (8) the tactile sensory threshold before surgery (patients with low sensory thresholds before BSSO experienced a higher degree of impairment after surgery compared with those with higher preoperative thresholds) [9, 17, 30, 35, 41]. Additionally, Terijoki-Oksa et al. reported that the continuous intraoperative monitoring of the sensory nerve action potential (SNAP) of the IAN showed that the medial opening (retraction of bone fragments on the inner side of the mandible) during the BSSO involves a high risk of IAN damage [17]. Patients with altered sensation were faced not only with unfamiliar sensory experience of their lips, chin, and mouth, but also had problems with facial function. Problems with function that are frequently reported include the following: drooling, undetected food particles remaining on the lip and chin, errors in speech articulation, dysaesthesia or pain upon touching the gingiva, often resulting in poor oral hygiene habits, cheek biting, difficulty with eating and kissing [9]. Ow et al. conducted a prospective clinical trial comparing the neurosensory function of the IAN after mandibular advancement surgery with BSSO and mandibular distraction osteogenesis (MDO). The results showed some degree of postoperative neurosensory disturbance for patients 1 year postoperatively after both types of surgery [7]. Despite the fact that there is less traumatic manipulation of the IAN with MDO than with BSSO, nerve function remained similar after both types of procedure. The infraorbital nerve (ION) is another cranial nerve that may be exposed to injury during orthognathic surgery procedures. Thygesen et al. found that subjective changes in somatosensory function after LeFort I osteotomy occurred in 7 to 60 % of patients, depending on the site of measurement at 12-month follow-up [44]. Changes in cutaneous, mucosal, and pulpal thresholds occurred as a result of LeFort I osteotomy, and significant side effects such as cutaneous numbness and hypersensitivity, as well as intraoral numbness in the facial and palatal gingiva, are associated with that procedure [44]. Furthermore, segmentation of the maxilla additionally decreased sensory function in the palate and gingiva [44]. Despite these sensory problems, many patients were satisfied with their surgical results and would recommend the surgery procedure to other patients needing a combined orthodontic surgical treatment [44].

Temporomandibular joint (TMJ) disorders represent the second most commolny described complication after orthognathic surgery (13.64 %). After surgery, patients may suffer from TMJ dysfunction, derangement of the condylar surface, condylar resorption, or malocclusion as a result of condylar sag [10, 11, 22, 33]. Consensus on the influence of orthognathic surgery on TMJ dysfunction has also not yet been achieved [33]. Some investigators have reported a favorable effect of orthognathic surgery on TMJ dysfunction; however, other studies did not show an improvement of TMJ symptoms, and TMJ function worsened in some patients [33]. Intraoral oblique ramus osteotomy with maxilla-mandibular fixation appears to be more favorable for TMJ than BSSO with rigid fixation, especially in patients with preoperative symptoms [24]. However, Nemeth et al. used a randomized clinical trial design, and calibrated examiners found no significant differences in TMJ signs or symptoms between wire and rigid fixation 2 years after surgery [6]. Diverse TMJ symptoms may occur after orthognathic surgery, ranging from intra-articular noise [53–55], pain, clicking, and crepitus, to condylar resorption [56]. Surgeons should be aware of the risk of condylar resorption, especially when the patient is a female and exhibits a high preoperative plane angle, small condyles (on panoramic X-rays), class II angle deformity requiring wide mandibular advancement, and a posteriorly inclined condylar neck [22, 56]. The first signs of condylar resorption are apparent 6 months or more after mandibular advancement and could develop up to 2 years postoperatively [22].

Condylar sag can be defined as an immediate or late change in position of the condyle in the glenoid fossa after the surgical establishment of preplanned occlusion and rigid fixation of the bone fragments, leading to changes in the occlusion [10, 11]. This condition is divided into central and peripheral categories, which are divided into peripheral condylar sag type I and peripheral condylar sag type II. This division is based on the relationship between the articular surfaces [11]. Possible risk factors include the following: (1) incorrect vector during condylar positioning; (2) an incomplete or green-stick split that prevents condylar seating; (3) muscular, ligamentous, or periosteal interference; 4) intra-articular hemorrhage or edema; and flexing the proximal segment while placing rigid fixation [11]. The most important part of surgery for avoiding such complications is the positioning of bony fragments and rigid fixation. Methods that help to cope with these challenges include the following: (1) intraoperative diagnosis [11]; (2) specific condylar positioning technique [11]; and (3) intraoperative awakening of the patient in a state of conscious analgo-sedation to examine passive and active movements of the mandible to create the correct occlusal relationship [10].

Hemorrhage after LeFort I surgery was described in 9.09 % evaluated articles. The most serious hemorrhage during or after Le Fort I osteotomy happens as a consequence of pterygomaxillary separation [19, 21]. The risk of arterial bleeding from the posterior maxilla usually arises from the descending palatine artery or less frequently from the maxillary artery and its branches. Serious hemorrhage from the pterygoid venous plexus occurs less frequently [19]. The patterns of fracture of the pterygoid plates in conventional pterygomaxillary dysjunction seem to have a great influence on the occurrence of bleeding. According to a trial by Regan et al., the tuberosity osteotomy technique reduces the likelihood of an unfavorable fracture of the pterygoid plates [19]. Based on the given studies, hemorrhage was indicated as the most common complication in maxillary surgery [12]. In contrast to the incidence of the manageable hemorrhage, the life-threatening postoperative hemorrhage after Le Fort osteotomy is rare and varies between an incidence of 0 and 0.7 % [21]. A combination of conservative and surgical treatment is initiated in most cases of life-threatening hemorrhage. Conservative treatment consists of controlling blood pressure and administering intravenous fluids and blood transfusion. The surgical approach includes simple nasal packing, revision osteotomy, and ligation of the branches of external carotid artery [14, 21].

Auditory tube function and hearing problems were citated in 6.82 % of articles. Some aural symptoms (tinnitus, fullness, otalgia) and auditory changes may occur as a consequence of surgical edema or lymphoedema and hematoma [38]. Nasotracheal intubation may also cause swelling of the soft tissues in the nasotracheal area, blocking the Eustachian tube, and precipitating middle ear effusion [38]. Decreased auditory function can most likely be assigned to Eustachian tube changes of orientation as a result of increased and changed muscular tension. The commonly accepted method of pathogenesis is the scarring or compromise of musculature that opens the auditory tube and ventilates the middle ear [28]. The most important muscle seems to be the tensor veli palatine muscle, which actively opens and closes the Eustachian tube [28]. Its mechanical impairment may lead to poor function of the tube and the loss of middle ear integrity [28]. The mean time of auditory changes and Eustachian tube functional evaluation follow-up was between 6–8 weeks [28, 42] and 6 months [38]. Hearing loss at 6–8 weeks postoperatively varies from 6 to 38 % [28, 38, 42]. Wong et al. found significantly less prevalence of hearing problems and middle ear dysfunction among Chinese patients [38]. This finding may be attributed to differences in the surgical approach and the fact that the Chinese population may be less susceptible to middle ear problems [38].

Infections reported in 6.82 % of evaluated articles occurred due to healing problems around miniplates and monocortical screws and were also observed as maxillary sinusitis or abscesses [14, 20, 23]. According to Alpha et al., disturbance of healing (DOH) occurred at a surprisingly high incidence (26 %) in the BSSO patient group, but more importantly, this group of patients showed a low incidence of hardware removal (6.5 %) [20]. Patients who underwent bimaxillary surgery had a lower incidence of DOH compared with those after isolated BSSO [20]. An explanation of this result could be the 23-h evaluation period and the administration of several (2–5) doses of intravenous antibiotics for such patients [20]. In this study, smoking patients with third molars and diabetics showed a higher incidence of DOH. The location of plates and screws relative to the inferior border of the mandible and impaired vascularization of the proximal segment may be factors contributing to the higher incidence of DOH [20]. Sinusitis aetiologies were reported to be related to the mechanical obstruction of drainage of the osteomeatal complex region. Anatomical abnormalities (presence of concha bullosa, septum deviation, paradoxal turbinate concha, malformation of uncinated process) may cause decreased drainage of the maxillary sinus. Maxillary osteotomy and long-term intubation can also block sinus drainage and lead to mucus stasis, which predisposes the patients to bacterial and/or fungal sinus infection [23]. The incidence of maxillary sinusitis as a postoperative complication after Le Fort I osteotomy ranges from 0.6 to 4.76 % [14, 23].

The term “bad split” described in the literature as an unfavorable or irregular fracture of the mandible can occur with an incidence that varies from 1 to 23 % [18]. Bad split can be provoked by the following: (1) an anatomically thin mandibular ramus; (2) a high mandibular lingual; (3) the presence of third molars; or even (4) by the inexperience or lack of attention of the surgeon [18]. Some authors suggested that the use of heavy osteotomes, twisting techniques, or the incomplete split of the inferior border of the mandible could be the main cause of bad splitting [18]. A study performed by Veras et al. showed statistical significance in the correlation between the age of the patient and the occurrence of a bad split [18].

In conclusion, we need to confirm the null hypothesis. There exist a large number of varied complications associated with orthognathic surgery procedures. Regardless, complications may occur after every surgery, and surgeons are obligated to minimize the risk of complications. The oral and maxillofacial surgeons, the orthodontist, and the operating team must prevent such complications during the preoperative, intraoperative, and postoperative periods to increase the safety of orthognathic surgery procedures. The permanent increase of surgery technique, methods of orthodontic treatment, and experience is absolutely needed. Despite during our research, we found many studies reporting complications in orthognathic surgery, the majority of obtained studies were case reports, case series, or reviews. These types of studies do not currently provide reliable evidence. Additionally, the critical appraisal of all included RCTs and CCTs resulted in only three studies that were assessed as having a low risk of bias. Therefore, more good quality RCTs and CCTs are needed to provide better evidence in this field.

### Limitations of the study

This SR exhibits some limitations. We did not discuss complications associated with purely anesthesiological procedures, such as perforation of the endotracheal tube, pulmonary edema, or cerebral hypoxia. We also did not discuss complications associated with the prevention of laboratory errors, poor team communication or those that could only be resolved by more general procedures (certification of operating room facilities), communication workshops, team building, etc. We were not able to compare the number of described (underestimated) complications with the total number of osteotomies ever performed in the world to relativize the problem of complications in orthognathic surgery.

## Electronic supplementary material

Below is the link to the electronic supplementary material.

Online Resource 1 Risk of bias assessment graph: review authors’ judgements about each risk of bias item for each included Randomized Clinical Trials (DOCX 23 kb)

Online Resource 2 Risk of bias assessment graph: review authors’ judgements about each risk of bias item for each included Clinical Trial (DOCX 25 kb)

ESM 3 (DOC 63 kb)

ESM 4 (PDF 1855 kb)

## References

[1] Ow A, Cheung LK (2009). Skeletal stability and complications of bilateral sagittal split osteotomies and mandibular distraction osteogenesis: an evidence-based review. J Oral Maxillofac Surg.

[2] Ruiz LP, Lara JC (2011). Facial nerve palsy following bilateral sagittal split ramus osteotomy for setback of the mandible. Int J Oral Maxillofac Surg.

[3] Chrcanovic BR, Custódio AL (2011). Optic, oculomotor, abducens, and facial nerve palsies after combined maxillary and mandibular osteotomy: case report. J Oral Maxillofac Surg.

[4] Rai KK, Shivakumar HR, Sonar MD (2008). Transient facial nerve palsy following bilateral sagittal split ramus osteotomy for setback of the mandible: a review of incidence and management. J Oral Maxillofac Surg.

[5] Piñeiro-Aguilar A, Somoza-Martín M, Gandara-Rey JM, García-García A (2011). Blood loss in orthognathic surgery: a systematic review. J Oral Maxillofac Surg.

[6] Nemeth DZ, Rodrigues-Garcia RC, Sakai S, Hatch JP, Van Sickels JE (2000). Bilateral sagittal split osteotomy and temporomandibular disorders: rigid fixation versus wire fixation. Oral Surg Oral Med Oral Pathol Oral Radiol Endod.

[7] Ow A, Cheung LK (2010). Bilateral sagittal split osteotomies versus mandibular distraction osteogenesis: a prospective clinical trial comparing inferior alveolar nerve function and complications. Int J Oral Maxillofac Surg.

[8] Essick GK, Phillips C, Turvey TA, Tucker M (2007). Facial altered sensation and sensory impairment after orthognathic surgery. Int J Oral Maxillofac Surg.

[9] Phillips C, Essick G, Blakey G, Tucker M (2007). Relationship between patients' perceptions of postsurgical sequelae and altered sensations after bilateral sagittal split osteotomy. J Oral Maxillofac Surg.

[10] Politi M, Toro C, Costa F, Polini F, Robiony M (2007). Intraoperative awakening of the patient during orthognathic surgery: a method to prevent the condylar sag. J Oral Maxillofac Surg.

[11] Reyneke JP, Ferretti C (2002). Intraoperative diagnosis of condylar sag after bilateral sagottal split Ramus osteotomy. Br J Oral Maxillofac Surg.

[12] Gunaseelan R, Anantanarayanan P, Veerabahu M, Vikraman B, Sripal R (2009). Intraoperative and perioperative complications in anterior maxillary osteotomy: a retrospective evaluation of 103 patients. J Oral Maxillofac Surg.

[13] Kahnberg KE, Ridell A (1987). Transposition of the mental nerve in orthognathic surgery. J Oral Maxillofac Surg.

[14] Kramer FJ, Baethge C, Swennen G, Teltzrow T, Schulze A (2004). Intra- and perioperative complications of the LeFort I osteotomy: a prospective evaluation of 1000 patients. J Craniofac Surg.

[15] Li KK, Stephens W (1996). Fractures of the atrophic, edentulous maxilla during Le Fort I osteotomy. Int J Oral Maxillofac Surg.

[16] Wittwer G, Adeyemo WL, Beinemann J, Juergens P (2012). Evaluation of risk of injury to the inferior alveolar nerve with classical sagittal split osteotomy technique and proposed alternative surgical techniques using computer-assisted surgery. Int J Oral Maxillofac Surg.

[17] Teerijoki-Oksa T, Jääskeläinen SK, Forssell K, Forssell H, Vähätalo K (2002). Risk factors of nerve injury during mandibular sagittal split osteotomy. Int J Oral Maxillofac Surg.

[18] Veras RB, Kriwalsky MS, Hoffmann S, Maurer P, Schubert J (2008). Functional and radiographic long-term results after bad split in orthognathic surgery. Int J Oral Maxillofac Surg.

[19] O'Regan B, Bharadwaj G (2007). Prospective study of the incidence of serious posterior maxillary haemorrhage during a tuberosity osteotomy in low level Le Fort I operations. Br J Oral Maxillofac Sur.

[20] Alpha C, O'Ryan F, Silva A, Poor D (2006). The incidence of postoperative wound healing problems following sagittal ramus osteotomies stabilized with miniplates and monocortical screws. J Oral Maxillofac Surg.

[21] Politis C (2012). Life-threatening haemorrhage after 750 Le Fort I osteotomies and 376 SARPE procedures. Int J Oral Maxillofac Surg.

[22] Kobayashi T, Izumi N, Kojima T, Sakagami N, Saito I (2012). Progressive condylar resorption after mandibular advancement. Br J Oral Maxillofac Surg.

[23] Pereira-Filho VA, Gabrielli MF, Gabrielli MA, Pinto FA, Rodrigues-Junior AL (2011). Incidence of maxillary sinusitis following Le Fort I osteotomy: clinical, radiographic, and endoscopic study. J Oral Maxillofac Surg.

[24] Hu J, Wang D, Zou S (2000). Effects of mandibular setback on the temporomandibular joint: a comparison of oblique and sagittal split ramus osteotomy. J Oral Maxillofac Surg.

[25] Gulses A, Aydintug YS, Sencimen M, Bayar GR, Acikel CH (2012). Evaluation of neurosensory alterations via clinical neurosensory tests following anterior maxillary osteotomy (Bell technique). Int J Oral Maxillofac Surg.

[26] Stewart TD, Sexton J (1987). Depression: a possible complication of orthognathic surgery. J Oral Maxillofac Surg.

[27] Neal CE, Kiyak HA (1991). Patient perceptions of pain, paresthesia, and swelling after orthognathic surgery. Int J Adult Orthodon Orthognath Surg.

[28] Yaghmaei M, Ghoujeghi A, Sadeghinejad A, Aberoumand D, Seifi M (2009). Auditory changes in patients undergoing orthognathic surgery. Int J Oral Maxillofac Surg.

[29] Teerijoki-Oksa T, Jääskeläinen SK, Soukka T, Virtanen A, Forssell H (2011). Subjective sensory symptoms associated with axonal and demyelinating nerve injuries after mandibular sagittal split osteotomy. J Oral Maxillofac Surg.

[30] Kuroyanagi N, Miyachi H, Ochiai S, Kamiya N, Kanazawa T, Nagao T, Shimozato K (2012). Prediction of neurosensory alterations after sagittal split ramus osteotomy. Int J Oral Maxillofac Surg.

[31] Gianni AB, D'Orto O, Biglioli F, Bozzetti A, Brusati R (2002). Neurosensory alterations of the inferior alveolar and mental nerve after genioplasty alone or associated with sagittal osteotomy of the mandibular ramus. J Craniomaxillofac Surg.

[32] Ylikontiola L, Kinnunen J, Laukkanen P, Oikarinen K (2000). Prediction of recovery from neurosensory deficit after bilateral sagittal split osteotomy. Oral Surg Oral Med Oral Pathol Oral Radiol Endod.

[33] Onizawa K, Schmelzeisen R, Vogt S (1995). Alteration of temporomandibular joint symptoms after orthognathic surgery: comparison with healthy volunteers. J Oral Maxillofac Surg.

[34] Gent JF, Shafer DM, Frank ME (2003). The effect of orthognathic surgery on taste function on the palate and tongue. J Oral Maxillofac Surg.

[35] Kobayashi A, Yoshimasu H, Kobayashi J, Amagasa T (2006). Neurosensory alteration in the lower lip and chin area after orthognathic surgery: bilateral sagittal split osteotomy versus inverted L ramus osteotomy. J Oral Maxillofac Surg.

[36] Beshkar M, Hasheminasab M, Mohammadi F (2013). Benign paroxysmal positional vertigo as a complication of orthognathic surgery. J Craniomaxillofac Surg.

[37] Hanzelka T, Foltán R, Pavlíková G, Horká E, Sedý J (2011). The role of intraoperative positioning of the inferior alveolar nerve on postoperative paresthesia after bilateral sagittal split osteotomy of the mandible: prospective clinical study. Int J Oral Maxillofac Surg.

[38] Wong LL, Samman N, Whitehill TL (2002). Are hearing and middle ear statuses at risk in Chinese patients undergoing orthognathic surgery?. Clin Otolaryngol Allied Sci.

[39] Schultze-Mosgau S, Krems H, Ott R, Neukam FW (2001). A prospective electromyographic and computer-aided thermal sensitivity assessment of nerve lesions after sagittal split osteotomy and Le Fort I osteotomy. J Oral Maxillofac Surg.

[40] Høgevold HE, Mobarak KA, Espeland L, Krogstad O, Skjelbred P (2001). Plate fixation of extra-oral subcondylar ramus osteotomy for correction of mandibular prognathism: clinical aspects and short term stability. J Craniomaxillofac Surg.

[41] Thygesen TH, Bardow A, Helleberg M, Norholt SE, Jensen J (2007). Risk factors affecting somatosensory function after sagittal split osteotomy. J Oral Maxillofac Surg.

[42] Barker GR (1987). Auditory tube function and audiogram changes following corrective orthognathic maxillary and mandibular surgery in cleft and non-cleft patients. Scand J Plast Reconstr Surg Hand Surg.

[43] Posnick JC, Al-Qattan MM, Stepner NM (1996). Alteration in facial sensibility in adolescents following sagittal split and chin osteotomies of the mandible. Plast Reconstr Surg.

[44] Thygesen TH, Bardow A, Norholt SE, Jensen J, Svensson P (2009). Surgical risk factors and maxillary nerve function after Le Fort I osteotomy. J Oral Maxillofac Surg.

[45] Al-Delayme R, Al-Khen M, Hamdoon Z, Jerjes W (2013). Skeletal and dental relapses after skeletal class III deformity correction surgery: single-jaw versus double-jaw procedures. Oral Surg Oral Med Oral Pathol Oral Radiol.

[46] Ji-Young L, Young-Kyun K, Pil-Young Y, Nam-Ki L, Jong-Wan K (2014). Evaluation of Stability After Orthognathic Surgery With Minimal Orthodontic Preparation: Comparison According to 3 Types of Fixation. J Craniofac Surg.

[47] Prazeres LDKT, Muniz YVS, Barros KMA, Gerbi MEM, Laureano Filho JR (2013). Effect of Infrared Laser in the Prevention and Treatment of Paresthesia in Orthognathic Surgery. J Craniof Surg.

[48] Calabria F, Sellek L, Gugole F, Trevisol L, Bertolasi L (2013). J Craniof Surg.

[49] Van der Vlis M, Dentino KM, Vervloet B, Padwa BL (2014). J Oral Maxillofac Surg.

[50] Wassmund M (1939). Lehrbuch der praktischen Chirurgie des Mundes und der Kiefer.

[51] Trauner R, Obwegeser H (1955). Zur Operationstechnik bei der Progenie und anderen Unterkieferanomalien. Dtsch Zahn Mund-u Kieferheilk.

[52] Piecuch JF, West RA (1979). Spontaneous pneumomediastinum associated with orthognathic surgery. A case report. Oral Surg Oral Med Oral Pathol.

[53] Bays RA, Bouloux GF (2003). Complications of orthognathic surgery. Oral Maxillofac Surg Clin North Am.

[54] Wolford LM, Reiche-Fischel O, Mehra P (2003). Changes in temporomandibular joint dysfunction after orthognathic surgery. J Oral Maxillofac Surg.

[55] Hori M, Okaue M, Hasegawa M, Harada D, Kamogawa D (1999). Worsening of pre-existing TMJ dysfunction following sagittal split osteotomy: a study of three cases. J Oral Sci.

[56] Hwang SJ, Haers PE, Sailer HF (2000). The role of a posteriorly inclined condylar neck in condylar resorption after orthognathic surgery. J Craniomaxillofac Surg.

