# Supplementary material for: Preoperative, intraoperative, and postoperative complications in orthognathic surgery: a systematic review
Source: Clin Oral Investig. 2015 Mar 26;19(5):969–77. doi: 10.1007/s00784-015-1452-1 (PMC4434857; doi:10.1007/s00784-015-1452-1)
Supplement: Supplementary file 1 — Risk of bias assessment graph: review authors’ judgements about each risk of bias item for each included Randomized Clinical Trials (DOCX 23 kb) [file 784_2015_1452_MOESM1_ESM.docx]

Online Resource 1. Risk of bias graph: review authors’ judgements about each risk of bias item for each included Randomized Controlled Trials

Selection bias (random sequence generation)

Performance bias (blinding of outcome assessment)

Attrition bias (incomplete outcome)

Detection bias (blinding of participants and personel)

Reportnig bias (selective reporting)

Overall judgment

| Essick et al (8) | + | ? | + | ? | + | ? |
| --- | --- | --- | --- | --- | --- | --- |
| Politi et al (10) | ? | ? | + | ? | + | ? |
| Philips et al (9) | + | ? | + | ? | + | ? |
| Ow et al (7) | + | ? | + | ? | + | ? |
| Nemeth et al (6) | + | ? | + | ? | + | ? |
| + low risk  - high risk  ? unclear risk | | | | | | |
