# Supplementary material for: Preoperative, intraoperative, and postoperative complications in orthognathic surgery: a systematic review
Source: Clin Oral Investig. 2015 Mar 26;19(5):969–77. doi: 10.1007/s00784-015-1452-1 (PMC4434857; doi:10.1007/s00784-015-1452-1)
Supplement: Supplementary file 2 — Risk of bias assessment graph: review authors’ judgements about each risk of bias item for each included Clinical Trial (DOCX 25 kb) [file 784_2015_1452_MOESM2_ESM.docx]

Online Resource 2. Risk of bias graph : review authors’ judgements about each risk of bias item for each included Clinical Trials

Selection bias

Performance bias

Attrition bias

Detection bias

Reporting bias

Overall judgement

| Kramer et al (14) | - | ? | + | ? | - | - |
| --- | --- | --- | --- | --- | --- | --- |
| Posnick et al (43) | ? | ? | + | ? | - | - |
| Barker (42) | - | - | + | ? | - | - |
| Thygesen et al (41) | + | + | + | + | + | + |
| Hogevold et al (40) | + | + | + | ? | + | ? |
| Schultze-Mosgau et al (39) | ? | + | + | ? | + | ? |
| Wong et al (38) | + | + | ? | ? | + | ? |
| O’Regan et al (19) | + | + | + | ? | + | ? |
| Hanzelka et al (37) | + | + | + | ? | + | ? |
| Veras et al (18) | + | + | + | ? | ? | ? |
| Teerijoki-Oksa et al (29) | + | + | + | ? | + | ? |
| Beshkar et al (36) | + | ? | ? | ? | + | ? |
| Kobayashi et al (22) | + | + | + | ? | ? | ? |
| Wittwer et al (16) | ? | ? | ? | - | - | - |
| Gent et a (34) | - | + | - | ? | + | - |
| Onizawa et al (33) | + | + | + | ? | ? | ? |
| Ylikontiola et al (32) | ? | + | + | ? | + | ? |
| Gianni et al (31) | ? | + | + | ? | + | ? |
| Li et al (15) | + | ? | + | ? | ? | ? |
| Kuroyanagi et al (30) | + | + | + | + | + | + |
| Teerijoki-Oksa et al (17) | ? | + | + | ? | + | ? |
| Yaghmaei et al (28) | + | ? | + | ? | + | ? |
| Neal et al (27) | + | + | + | + | + | + |
| Kahnberg et al (13) | - | ? | ? | - | - | - |
| Stewart et al (26) | ? | + | ? | ? | + | ? |
| Gunaseelan et al (12) | - | + | - | - | - | - |
| Gulses et al (25) | + | + | ? | ? | + | ? |
| Hu et al (24) | + | ? | + | ? | + | ? |
| Pereira-Filho et al (23) | - | + | + | ? | + | - |
| Kobayashi et al (35) | - | - | ? | ? | + | - |
| Politis (21) | ? | ? | + | - | + | - |
| Alpha et al (20) | + | + | + | ? | + | ? |
| Thygesen et al (44) | + | + | + | ? | + | ? |
| Reyneke et al (11) | ? | + | + | - | + | - |
| Al.-Delayme et al (45) | ? | + | + | ? | + | ? |
| Ji-Young L et al. (46) | ? | ? | + | ? | + | ? |
| Prazeres LDKT et al. (47) | ? | + | + | ? | + | ? |
| Calabria F et al. (48) | ? | + | + | ? | + | ? |
| Van der Vlis M et al. (49) | + | ? | + | ? | + | ? |
| + low risk  - high risk  ? unclear risk | | | | | | |
