# Supplementary material for: Preoperative, intraoperative, and postoperative complications in orthognathic surgery: a systematic review
Source: Clin Oral Investig. 2015 Mar 26;19(5):969–77. doi: 10.1007/s00784-015-1452-1 (PMC4434857; doi:10.1007/s00784-015-1452-1)
Supplement: Supplementary file 4 — (PDF 1855 kb) [file 784_2015_1452_MOESM4_ESM.pdf]

AMERICAN JOURNAL EXPERTS

EDITORIAL CERTIFICATE

This document certifies that the manuscript listed below was edited for proper English language, grammar, punctuation, spelling, and overall style by one or more of the highly qualified native English speaking editors at American Journal Experts.

Manuscript title:

Preoperative, intraoperative and postoperative complications in orthognathic surgery: A systematic review.

Authors:

Jędrzejewski M, Smektała T, Sporniak-Tutak K, Olszewski R

Date Issued:

December 22, 2014

Certificate Verification Key:

F783-222B-2283-F8EA-A477

This certificate may be verified at www.aje.com/certificate. This document certifies that the manuscript listed above was edited for proper English language, grammar, punctuation, spelling, and overall style by one or more of the highly qualified native English speaking editors at American Journal Experts. Neither the research content nor the authors' intentions were altered in any way during the editing process. Documents receiving this certification should be English-ready for publication; however, the author has the ability to accept or reject our suggestions and changes. To verify the final AJE edited version, please visit our verification page. If you have any questions or concerns about this edited document, please contact American Journal Experts at support@aje.com.

American Journal Experts provides a range of editing, translation and manuscript services for researchers and publishers around the world. Our top-quality PhD editors are all native English speakers from America's top universities. Our editors come from nearly every research field and possess the highest qualifications to edit research manuscripts written by non-native English speakers. For more information about our company, services and partner discounts, please visit www.aje.com.
